# Supplementary material for: Developing a model to implement marker-assisted selection for root-knot nematode resistance in common bean
Source: G3 (Bethesda). 2025 Nov 7;15(12):jkaf221. doi: 10.1093/g3journal/jkaf221 (PMC12693622; doi:10.1093/g3journal/jkaf221)
Supplement: jkaf221_Supplementary_Data [file jkaf221_supplementary_data.zip › Supplementary_Figures_G3-2025-405679.docx]

**Supplementary Figures**

**Supplementary Figure 1:** Frequency distribution of phenotypic traits in the F_2:3_ families. **(a)** Egg Mass (EM) **(b)** Root-Galling Index (GI)**(c)** Root Dry Mass (RM)

**Supplementary Figure 2:** Correlation matrix of phenotypic traits showing the relationships between root mass (RM), egg mass (EM), and gall index (GI). The circle size represents the strength of the correlation, with numerical values indicating correlation coefficients.


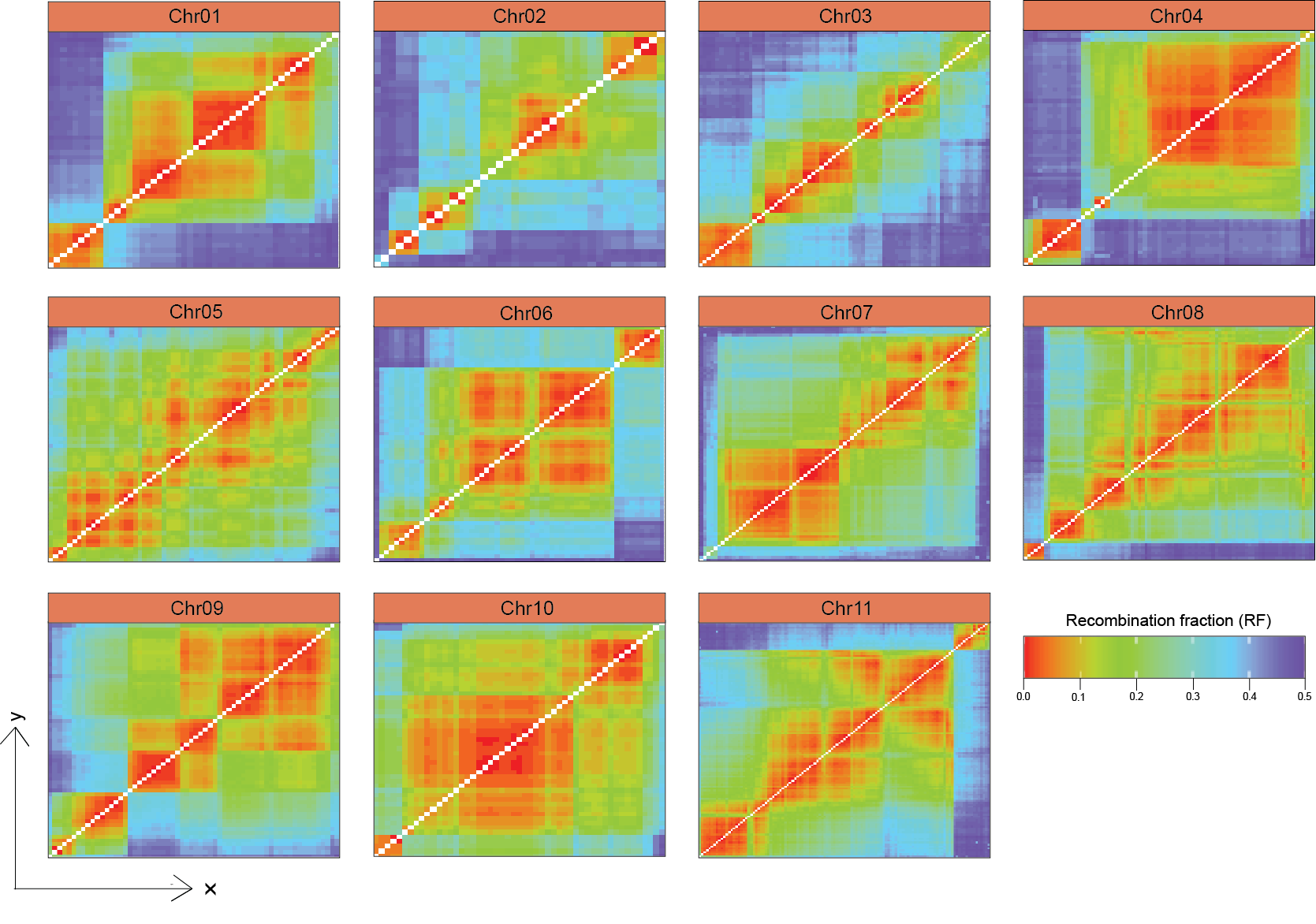


**Supplementary Figure 3:** Heatmaps displaying the recombination fraction between markers in 11 chromosomes.


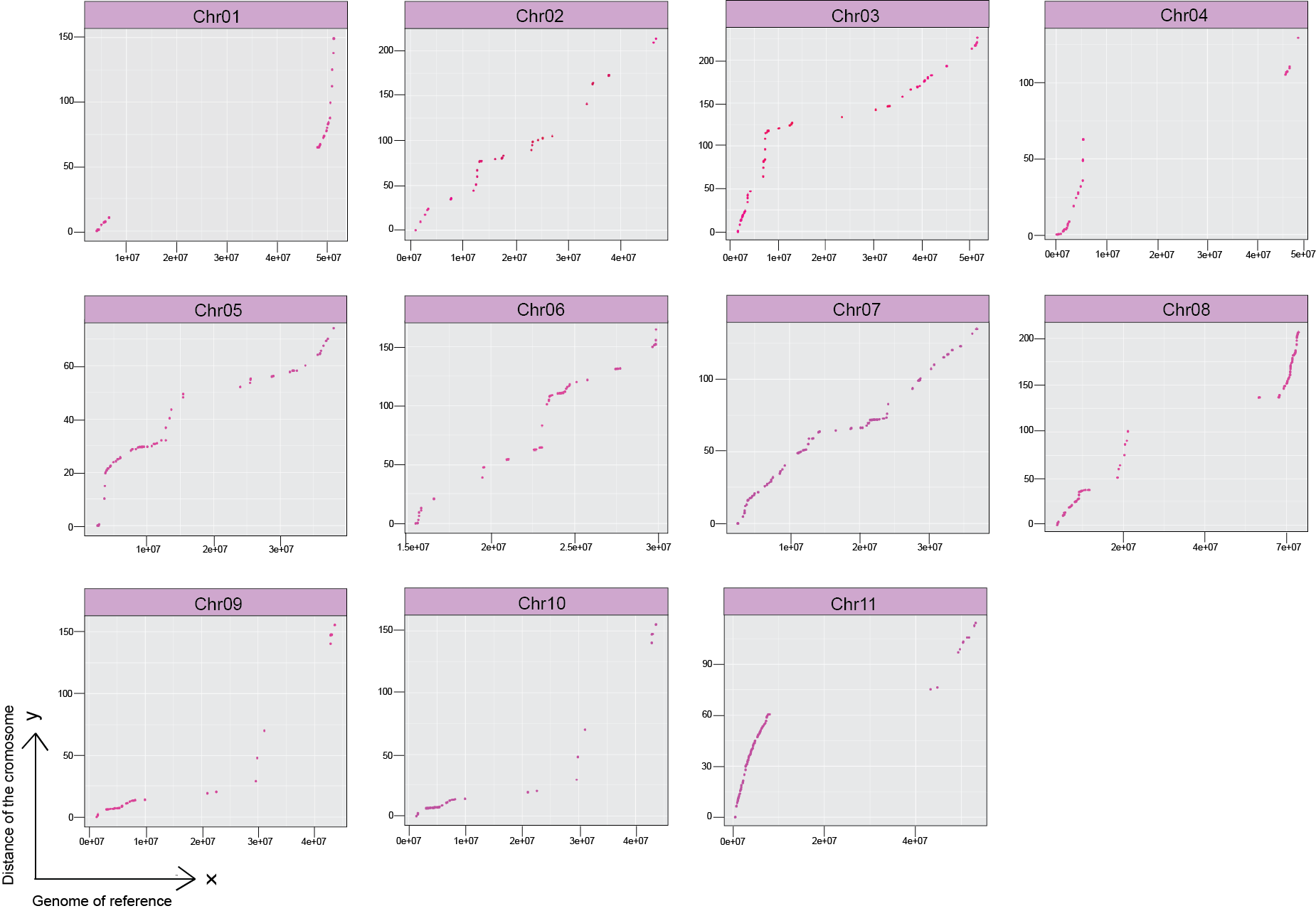


**Supplementary Figure 4:** Relationship between the genetic map and the common bean reference genome. The scatter plots depict the relationship between the physical positions of markers on the reference genome (x-axis) and their corresponding genetic map positions in centiMorgans (y-axis) for each chromosome (Pv01 to Pv11). Each point represents a marker, and the alignment along the diagonal indicates the consistency of the genetic map with the reference genome. Gaps show a possible centromere region.


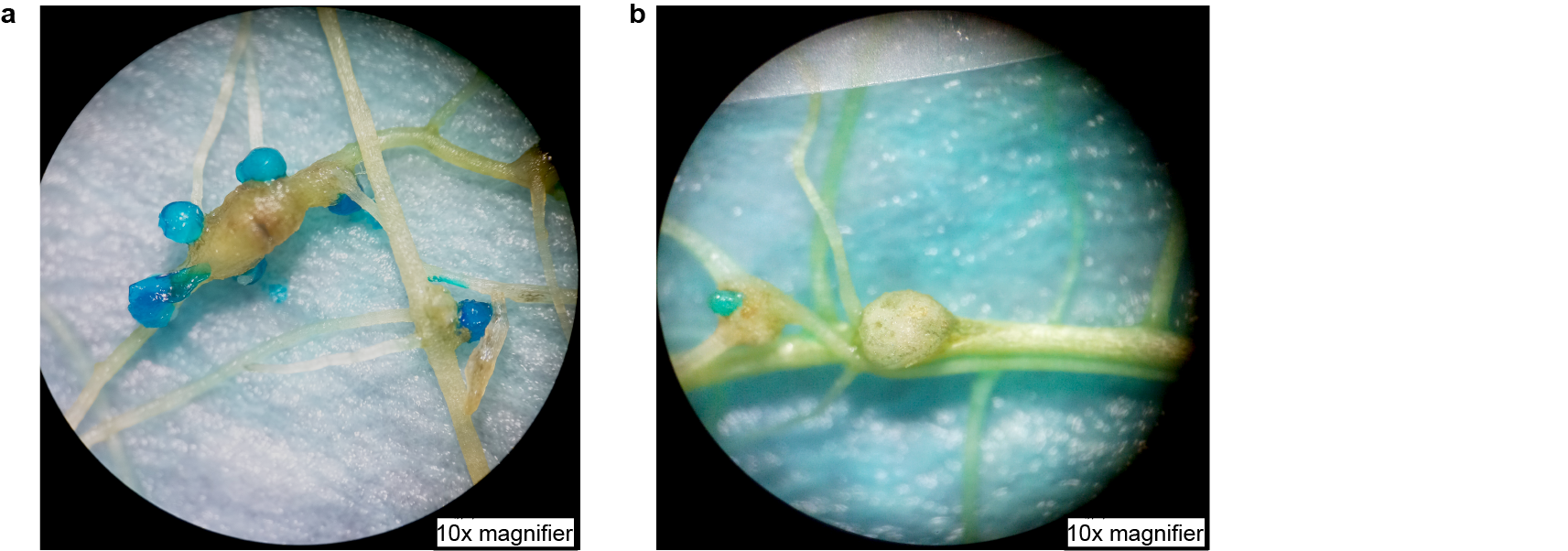


**Supplementary Figure 5:** Phenotypic evaluation of root-knot nematode (RKN) infection in common bean.  **(a)** Panel shows an egg mass (EM) stained with eriglaucine, highlighting RKN oviposition on the root surface. **(b)** Shows a root gall, an indicator of nematode-induced tissue hypertrophy. Both traits, egg mass (EM) and root-gallong index (RI), were assessed as part of the phenotyping to evaluate resistance levels among genotypes. Images captured under a 10x magnifier.
